# Tupanvirus-infected amoebas are induced to aggregate with uninfected cells promoting viral dissemination

**DOI:** 10.1038/s41598-018-36552-4

**Published:** 2019-01-17

**Authors:** Graziele Oliveira, Lorena Silva, Thiago Leão, Said Mougari, Flávio Guimarães da Fonseca, Erna Geessien Kroon, Bernard La Scola, Jônatas Santos Abrahão

**Affiliations:** 1 Departamento de Microbiologia, Instituto de Ciências Biológicas, Universidade Federal de Minas Gerais, Belo Horizonte, Minas Gerais 31270-901 Brazil; 2 URMITE, Aix Marseille Université, UM63, CNRS 7278, IRD 198, INSERM 1095, IHU - Méditerranée Infection, AP-HM, 19-21 boulevard Jean Moulin, 13005 Marseille, France

## Abstract

The discovery of giant viruses in the last years has fascinated the scientific community due to virus particles size and genome complexity. Among such fantastic discoveries, we have recently described tupanviruses, which particles present a long tail, and has a genome that contains the most complete set of translation-related genes ever reported in the known virosphere. Here we describe a new kind of virus-host interaction involving tupanvirus. We observed that tupanvirus-infected amoebas were induced to aggregate with uninfected cells, promoting viral dissemination and forming giant host cell bunches. Even after mechanical breakdown of bunches, amoebas reaggregated within a few minutes. This remarkable interaction between infected and uninfected cells seems to be promoted by the expression of a mannose receptor gene. Our investigations demonstrate that the pre-treatment of amoebas with free mannose inhibits the formation of bunches, in a concentration-dependent manner, suggesting that amoebal-bunch formation correlates with mannose receptor gene expression. Finally, our data suggest that bunch-forming cells are able to interact with uninfected cells promoting the dissemination and increase of tupanvirus progeny.

## Introduction

The recent discovery of tupanvirus, one of the largest and most complex viruses isolated to date, has reinforced the structural and genomic complexity of the giant viruses^1^. Tupanviruses have been isolated from soda lakes, known as an extreme aquatic environments, and from ocean sediments collected at a depth of 3000 meters (m)^1,2^. Phylogenetic analyses have shown the clustering of the tupanvirus with members of the family *Mimiviridae*. However, there are many peculiarities that make the tupanviruses unique entities in the known virosphere. Since its first observation, tupanviruses showed remarkable morphological characteristics; it has optically visible particles that average about 1.2 µm in size and can reach lengths up to 2.3 µm^1^. Tupanvirus has the largest host range described so far among amoebal-infecting giant viruses and can causes a shutdown of host rRNA that is likely related to host-nucleolus degradation^1,3–9^. The tupanviruses replication cycle is similar to those for other mimiviruses, in which viral particles attach to the host-cell surface and enter through phagocytosis. The viral inner membrane then fuses with the phagosome membrane, releasing the genome. A viral factory (VF) is formed, where particle morphogenesis occurs; the cycle ends with cell lysis and the release of progeny viruses^1,10^.

The study of the tupanvirus genome further aroused the interest of virologists, not only due of its large size (~1.5 Mb), but also because these viruses show the largest translational apparatus described. It is composed of up to 70 tRNA, 20 aminoacyl-tRNA synthetases (aaRS), 11 factors associated with translation, and factors related to tRNA/mRNA maturation and ribosome protein modification^1^. In addition to the robust translation apparatus, tupanvirus also contains a gene encoding mannose-specific lectin, also called mannose-binding protein (MBP)^1^. Interestingly, previous studies revealed that *Acanthamoeba castellanii* expresses an MBP and that free-mannose can inhibit the adhesion of *A. castellanii* to surfaces, suggesting that the MBP plays a role in the pathogenesis of *Acanthamoeba* infection^11–16^.

In this work, we describe that tupanviruses induces the expression of cellular and viral mannose receptor genes, which are well known for promoting the adhesion of amoebas to other cells. Tupanvirus promotes the formation of large bunches of infected and non-infected cells. In this context, we have hypothesized that infected cells act like “zombies,” controlled by tupanviruses, searching and attaching to uninfected cells, and improving the chances of the newly formed viral progeny to find a new host cell in which to propagate.

## Materials and Methods

### Cell culture, viral production and titration

*Acanthamoeba castellanii* cells (ATCC 30010) were cultivated in peptone-yeast extract with glucose (PYG) medium supplemented with 25 mg/ml amphotericin B (Fungizone; Cristalia, São Paulo, Brazil), 500 U/ml penicillin (Schering-Plough, Brazil) and 50 mg/ml gentamicin (Schering-Plough, Brazil). Cell culture flasks (175 cm^2^) containing 7 × 10^6^ *A. castellanii* cells were infected with tupanvirus strain soda lake at an multiplicity of infection (M.O.I) of 0.1 and incubated at 32 °C. After the appearance of a cytopathic effect and evidence of cell lysis, the cells and supernatants were collected and the viruses were purified through ultracentrifugation with a 22% sucrose cushion at 36,000 × g for 30 min. After viral purification, the virus titers were determined using the endpoint method^17,18^.

### Cytopathic effect and cycle characterization

**Figure 1:**
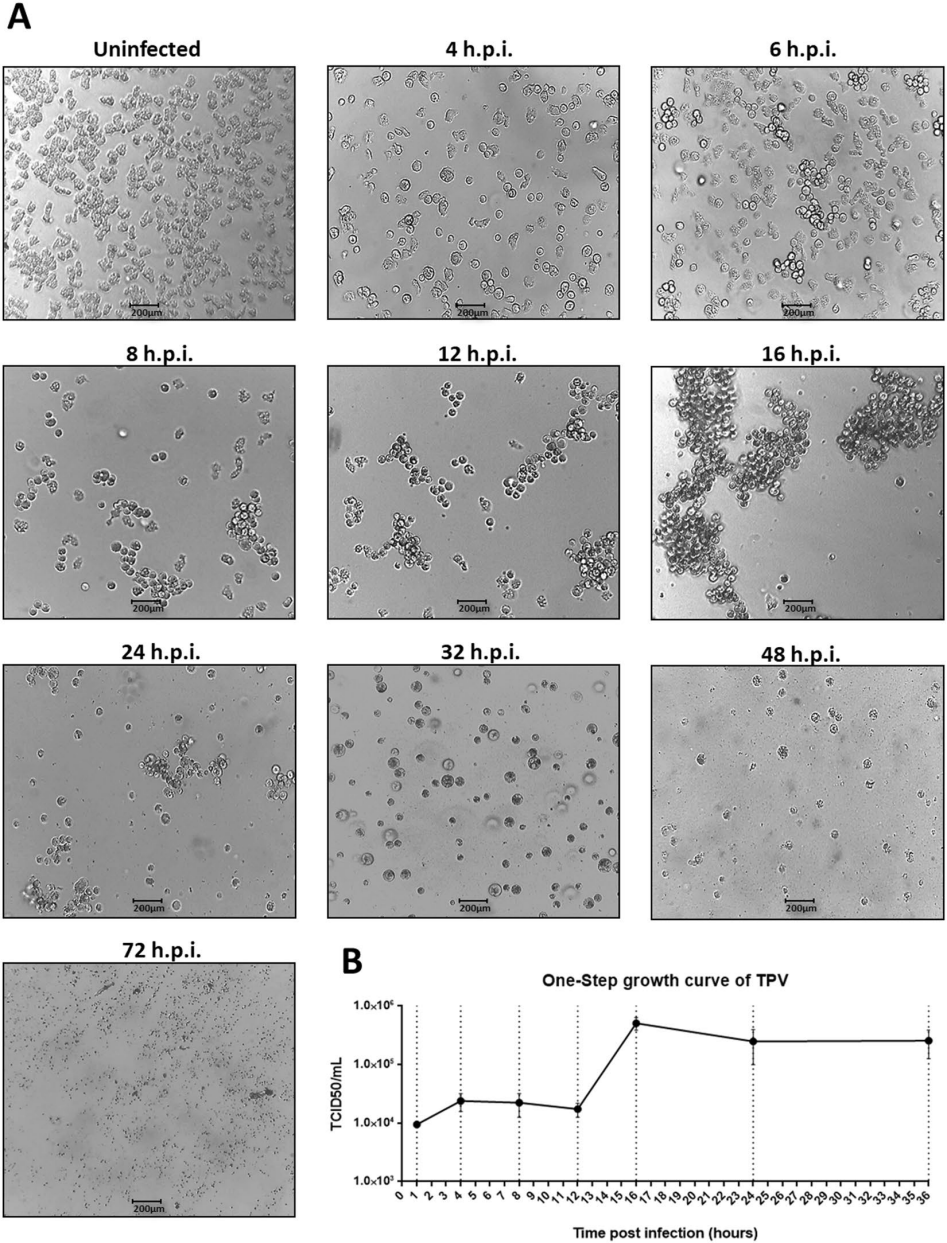
Characterization of tupanvirus’ cytopathic effect: formation of bunches. (**A**) *A. castellanii* monolayer was infected by tupanvirus using an M.O.I. of 10 and visualized by light microscopy. The induced cytopathic effects (CPEs) were observed by cell rounding at approximately 4 h.p.i. At approximately 6 h.p.i. the agglomeration of cells culminating in the formation of a characteristic effect, referred to as “bunches,” was observed. The presence of bunches becames more evident at 8, 12 and 16 h.p.i., when almost all the cells are clustered, forming large bunches. Cell lysis becomes more evident at 24 h.p.i. At about 32 h.p.i. a disaggregation of bunches was observed. The later timepoints are characterized by cell lysis (48 h.p.i.) and a large amount of viral particles dispersed throughout the medium (72 h.p.i.). Scale bar, 200 µm. (**B**) Tupanvirus one-step growth curve at an M.O.I. of 10. Error bars indicate standard deviation.

To investigate the cytopathic effect of tupanvirus in *A. castellanii* cells by optical microscopy, 25 cm^2^ cell culture flasks containing 1 × 10^6^ *A. castellanii* cells were infected with tupanvirus at an M.O.I of 0.01 and 10, incubated at 32 °C and observed at different timepoints post-infection for 72 hours. The *A. castellanii* cells were viewed under an optical microscopy at 0, 1, 2, 3, 4, 6, 8, 10, 12, 14, 16, 24, 32, 48 and 72 hours post-infection (h.p.i), and the more relevant times were presented. A infection control was developed using acanthamoeba polyphaga mimivirus (APMV), in which the same conditions were used at an MOI of 10. Uninfected *A. castellanii* cells (control) was also observed. A one-step growth curve was constructed using 25 cm^2^ flasks in duplicate at an M.O.I of 10 (Fig. 1B). At different timepoints, the infected *A. castellanii* cells and supernatants were collected, tittered and calculated using the end point method.

For immunofluorescence, *A. castellanii* cells were infected with tupanvirus at an M.O.I 10 and at 0 (immediately after contact between tupanvirus and *A. castellanii*), 1, 8, 12, 16 and 24 h.p.i., approximately 2 × 10^5^ cells were collected and centrifuged at 800 × g for 10 min. The pellet was resuspended in 50 µL of Page’s amoebae saline (PAS), and the cells were attached to a slide via cytospin and fixed in methanol for 15 min. After fixation, cells were incubated with 3% bovine serum albumin (BSA)-PAS for 30 min, followed by three rinses with 0.1% PAS-Tween. Cells were then stained with polyclonal anti-tupanvirus whole particle antibody produced in mouse (1:400 diluted in 3% BSA-PAS) for 1 h at 37 °C, followed by three rinses with PAS-Tween 0.1%. After a 1 h incubation with an anti-mouse secondary antibody (1:400 diluted in 3% BSA-PAS), one drop of 0.01% Evans Blue (Sigma), which was sufficient to cover the cells, was added and incubated for 15 min at 37 °C, followed by three rinses with PAS-Tween 0.1%. Uninfected cells (control) were also fixed and prepared as described. Fluorescently labeled cells were observed using an Axio Imager Z2-Apotome 2 microscope (Zeiss). The Zen Lite software from Zeiss microscopy was used for image processing.

For transmission electron microscopy (TEM), the cells were pelleted for 10 min at 800 × g. The pellet was washed twice with 0.1 M phosphate buffer (pH 7.4) and fixed with 2.5% glutaraldehyde in 0.1 M phosphate buffer for 1 h at room temperature. The pellet was then washed twice with 0.1 M phosphate buffer and suspended in the same buffer. After, amoebas were embedded in Epon resin the using a standard method, as follows: 2 h of fixation in 2% osmium tetroxide, five washes in distilled water, overnight incubation in uranyl acetate 2% at 2–8 °C, two washes in distilled water, 10 min dehydration in increasing ethanol concentrations (35, 50, 70, 85, 95 and 100%), 20 min incubation in acetone and embedding in EPON resin. Ultrathin sections were subsequently analyzed under TEM (Spirit Biotwin FEI-120 kV).

For scanning electron microscopy (SEM) assays, 50 µL of supernatant was added to round glass coverslips coated with poly-L-lysine and fixed with 2.5% glutaraldehyde in 0.1 M cacodylate buffer for 1 h at room temperature. The samples were washed three times with 0.1 M cacodylate buffer and post-fixed with 1% osmium tetroxide for 1 h at room temperature. After a second fixation, the samples were washed three times with 0.1 M cacodylate buffer and immersed in 0.1% tannic acid for 20 min. The samples were then washed in cacodylate buffer and dehydrated for 10 min by serial passages in ethanol solutions (35, 50, 70, 85, 95 and 100%). Samples were subsequently subjected to critical point drying using CO_2_, placed in stubs and metalized with a 5 nm gold layer. The analyses were completed using SEM (FEG Quanta 200 FEI).

### Mannose binding protein transcripts analyses and mannose assays

To evaluate the effect of mannose during tupanvirus infection, 1 × 10^6^ *A. castellanii* cells were infected with tupanvirus at an M.O.I of 10 in PAS medium containing different concentrations of alpha-D-mannopyranoside (25, 50, 100, 400, 600 and 1000 mM) and incubated at 32 °C. The cells were observed under an optical microscope at 8 h.p.i. In addition, the culture flasks were divided (2.5 cm^2^/field) and the counting of bunches was performed under an optical microscope. Cells infected by tupanvirus in PAS medium without mannose and PAS medium with 600 mM of mannose were titrated at 24 h.p.i. As controls, uninfected *A. castellanii* cells either cultivated in PAS medium without mannose or PAS medium containing 600 mM of mannose were used. To investigate the role of the mannose in the expression pattern of the *A. castellanii* and tupanvirus mannose binding protein genes, total RNA was purified from 5 × 10^5^ *A. castellanii* cells that had been infected with tupanvirus (M.O.I of 10) in the presence or absence of mannose (600 mM) at 1, 2, 4, 6, or 8 h.p.i. using the RNAeasy Mini Kit (Qiagen). Uninfected *A. castellanii* cells were used as controls. An control of infection using APMV was used to evaluate the expression of cellular MBP in amoebae infected with APMV, total RNA was purified from 5 × 10^5^ *A. castellanii* cells that had been infected with APMV (M.O.I of 10) at 1, 2, 4, 6, or 8 h.p.i. The samples were previously treated with DNAse (Invitrogen) and reverse transcribed using M-MLV Reverse Transcriptase (200 U/L; Thermo Fisher Scientific), according to the manufacturer’s instructions. The resulting cDNAs were used as a template in a StepOne thermocycler (Applied Biosystems) quantitative polymerase chain reaction (qPCR) assay to target the mannose receptor genes of the *A. castellanii* using oligo-nucleotide primers sequences Forward MBP AC _ CCAGTTCAATACCACCGGCT and Reverse MBP AC _ GTGGGGCAGTCCTTGTAGTC, tupanvirus (Forward MBP TPV _ ACGATTCAAGCCAGACACAA and Reverse MBP TPV _ GAGGAGTACCTTGCCCTGTTG) and APMV (Forward MBP APMV _ ACCCGCACCAGAAAGTCAAT and Reverse MBP APMV _ GGAGTCGGATTTGACGGTGT). The accession numbers of the genomes used to design the primers were AY604039.1, KY523104.1 and NC_014649.1 respectively. The thermal cycling conditions used were: one cycle at 95 °C for 10 min, 40 cycles at 95 °C for 10 sec and 60 °C for 40 sec; a melting curve analysis at 95 °C for 15 sec and 58 °C for 15 sec, and a final cycle was completed at 95 °C for 15 sec. The results were applied to the 2^−(Δct)^ method and analyzed by StepOne software. The assays were carried out in biological duplicate. Graphs were created using GraphPad Prism software (v7.0 for Windows).

### Evaluation of the interaction between uninfected amoebas and bunches

To evaluate whether bunches are able to interact with uninfected cells, 1 × 10^6^ *A. castellanii* cells in 25 cm^2^ culture flasks were infected with tupanvirus (M.O.I. of 10) and at 16 h.p.i. the bunches were collected and used to inoculate uninfected *A. castellanii* cells in suspension in a separate 25 cm^2^ culture flasks. After incubation for 30 min at 32 °C the non-adherent cells were removed from the culture flasks and the cells that remained adhered were counted. As a control, 16 h.p.i. APMV-infected amoebae were used in the same procedure. The experiment was performed in triplicate. The results were plotted on a column graph, and the statistical significance was calculated using a two-tailed Student’s t test, performed using Graph Pad Prism. In addition, the uninfected cells that interacted with bunches and were carried by them (the cells that did not adhere to the culture flasks again) were assayed using IF and SEM, as described above. To evaluate the importance of bunches with fresh cells for propagation of tupanvirus, 7 × 10^6^ *A. castellanii* cells were infected (M.O.I of 10) and at 16 h.p.i. the medium was removed; the bunches were washed and used to infect new *A. castellanii* cells and as a control, the bunches were also added to a flask containing fresh medium. At 36 h.p.i. the supernatants were collected and titrated as described above. Graphs were constructed using GraphPad Prism software (v7.0 for Windows).

## Results and Discussion

### Characterization of the tupanvirus cytopathic effect: a giant virus inducing cell aggregation

In order to characterize the cytopathic effect (CPE) triggered by tupanvirus, *A. castellanii* cells were infected at an M.O.I. of 10 and observed at up to 72 h.p.i. The cells became rounded and lost their adherence; this effect became more evident at 4 h.p.i. (Fig. 1A). Interestingly, tupanvirus exhibits a peculiar CPE that is characterized by amoebae aggregates, called “bunches.” The formation of bunches starts at approximately 6 h.p.i. and becomes more evident at 8, 12 and 16 h.p.i., when almost all the cells are seen incorporating into large bunches (Fig. 1A). From 24 h.p.i., despite the presence of bunches, cell lysis is more evident and is concomitant with the reduction of bunches size. At 32 h.p.i., the complete disaggregation of bunches was observed, and at 48–72 h.p.i. the majority of amoebae were lysed (Fig. 1A). The formation of bunches was also observed when a low M.O.I. (0.01) was used, although there was a temporal delay in bunch appearance (Suppl. Fig. 1). The CPE triggered by tupanvirus is unlike that of the other mimivirus, as well as other known giant amoebal viruses. Acanthamoeba polyphaga mimivirus, the prototype of the genus *Mimivirus*, triggers rounding and lysis in *A. castellanii* cell cultures, being a CPE commonly described for other giant viruses (M.O.I. of 10) (Suppl. Fig. 2).

**Figure 2:**
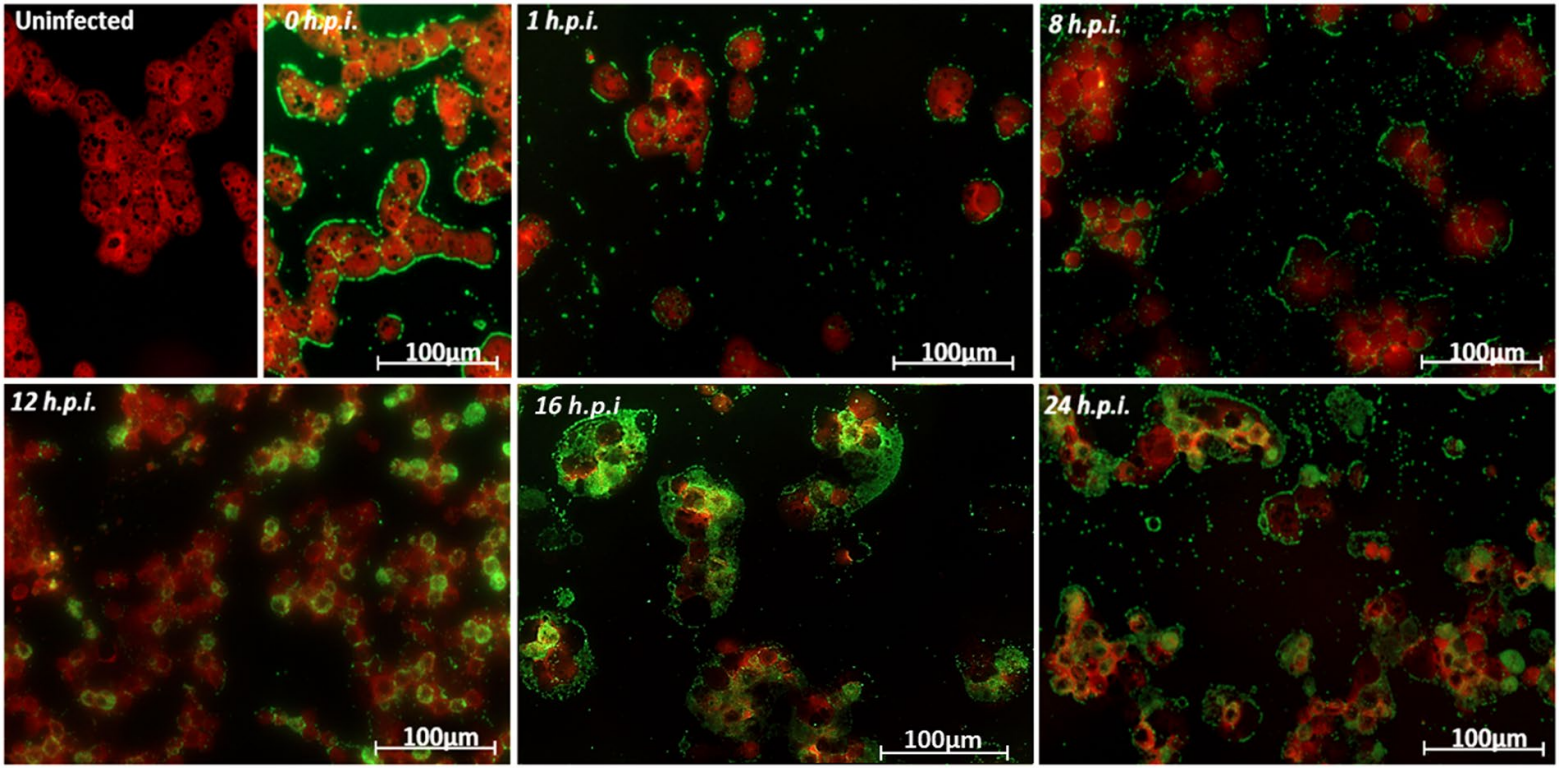
Characterization of the tupanvirus replication cycle in *A. castellanii* by IF. *A. castellanii* monolayer was infected by tupanvirus at an M.O.I. of 10 and visualized by IF. At early timepoints, tupanvirus particles are observed attaching to the amoebae surfaces, even after th cell washing step was done. The VF forms between 8 and 12 h.p.i. At 16 and 24 h.p.i. the *A. castellanii* cytoplasm is filled with viral particles and cell lysis is observed at 24 h.p.i. (at which time the cytoskeleton of lysed amoebas is visualized, although these cells remain adhered). The viral particles are in green (anti-tupan particle antibody) and amoeba cytoskeleton in red (stained by Evans Blue). Scale bar, 100 µm.

**Figure 3:**
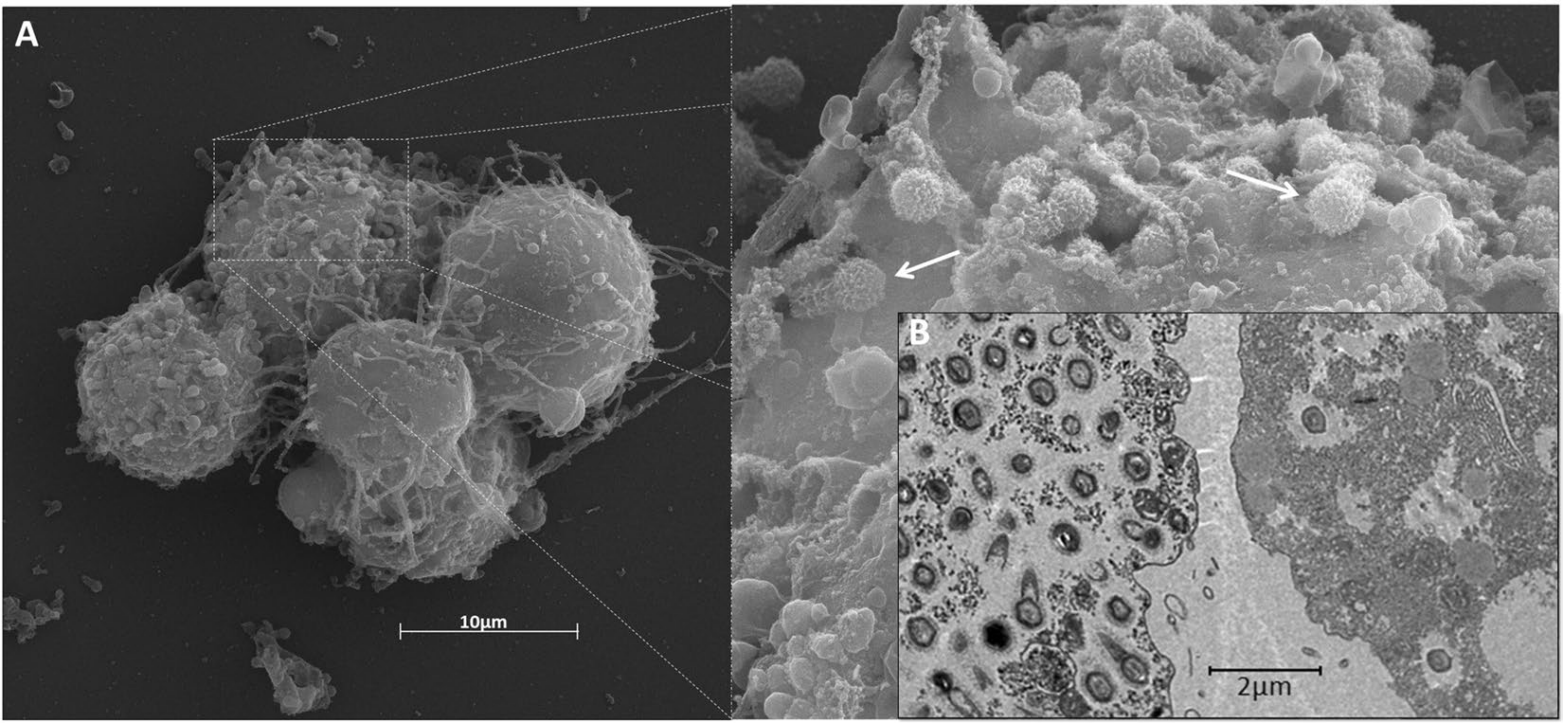
Detailed images of a 16 h.p.i. bunch. The bunches induced by tupanvirus in *A. castellanii* cells at different stages of infection were analyzed by: (**A**) Scanning electron microscopy and (**B**) transmission electron microscopy. White arrows indicate some tupanvirus particles being released from damaged amoebas. Scale bars, 2 µm and 10 µm.

Different timepoints during infection (at an M.O.I. of 10) were selected for an immunofluorescence assay (IF) and transmission/scanning electron microscopy (TEM and SEM). IF assays using anti-tupan antibody revealed that at 0 and 1 h.p.i. tupanvirus particles appeared attached to amoebas (Fig. 2), even after the cell washing step, suggesting virion fibrils early interactions with host-cells, as demonstrated for other mimiviruses^19^. VFs start to be detected by IF in amoebas’ cytoplasm using anti-tupanvirus whole-particle antibodies at 12 h.p.i. (Fig. 2). Curiously, even at high a M.O.I. it was not possible to observe a synchronous infection of all cells, something that was peculiarity related to tupanvirus and that needs further investigation. Remarkably, we observed amoebas with and without VFs or viral particles in the cytoplasm by IF, especially at 12 h.p.i., suggesting that early bunches are formed by the association of cells at different stages of the viral replication cycle (Fig. 2). Even at 16 h.p.i., when the viral titer reached its plateau (Fig. 1B) and the significant majority of cells already presented viral factories and particles (Fig. 2), we were able to find a very few cells with no detectable signs of tupanvirus by IF. By SEM (Fig. 3A) and TEM (Fig. 3B), it was possible at 16 h.p.i. to observe bunches of amoebas with damaged cells filled with or releasing tupanvirus particles, which were associating with cells with an intact plasma membrane and only a few particles inside them (Fig. 3).

### Early amoebal bunches reform after mechanical separation of cells

**Figure 4:**
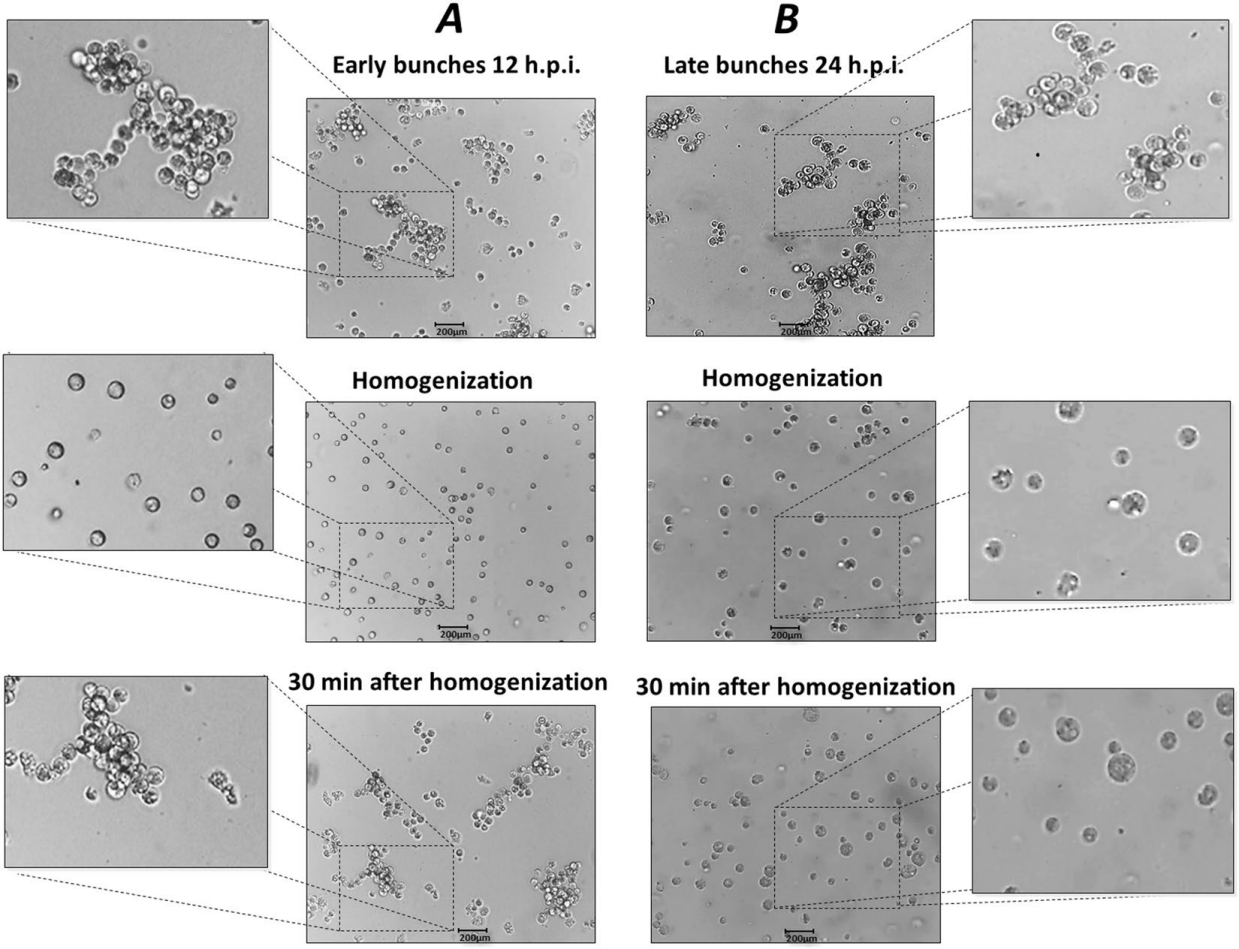
Early bunches re-aggregate after disassociation. *A. castellanii* cells infected with tupanvirus. Early (12 h.p.i.) and late (24 h.p.i.) bunches are shown, which were mechanically homogenized for cell disaggregation. Thirty minutes later, the cells were analyzed. (**A**) Re-aggregation of the early bunches 30 min after disassociation. (**B**) Re-aggregation of late bunches was not observed. Scale bar, 200 µm.

**Figure 5:**
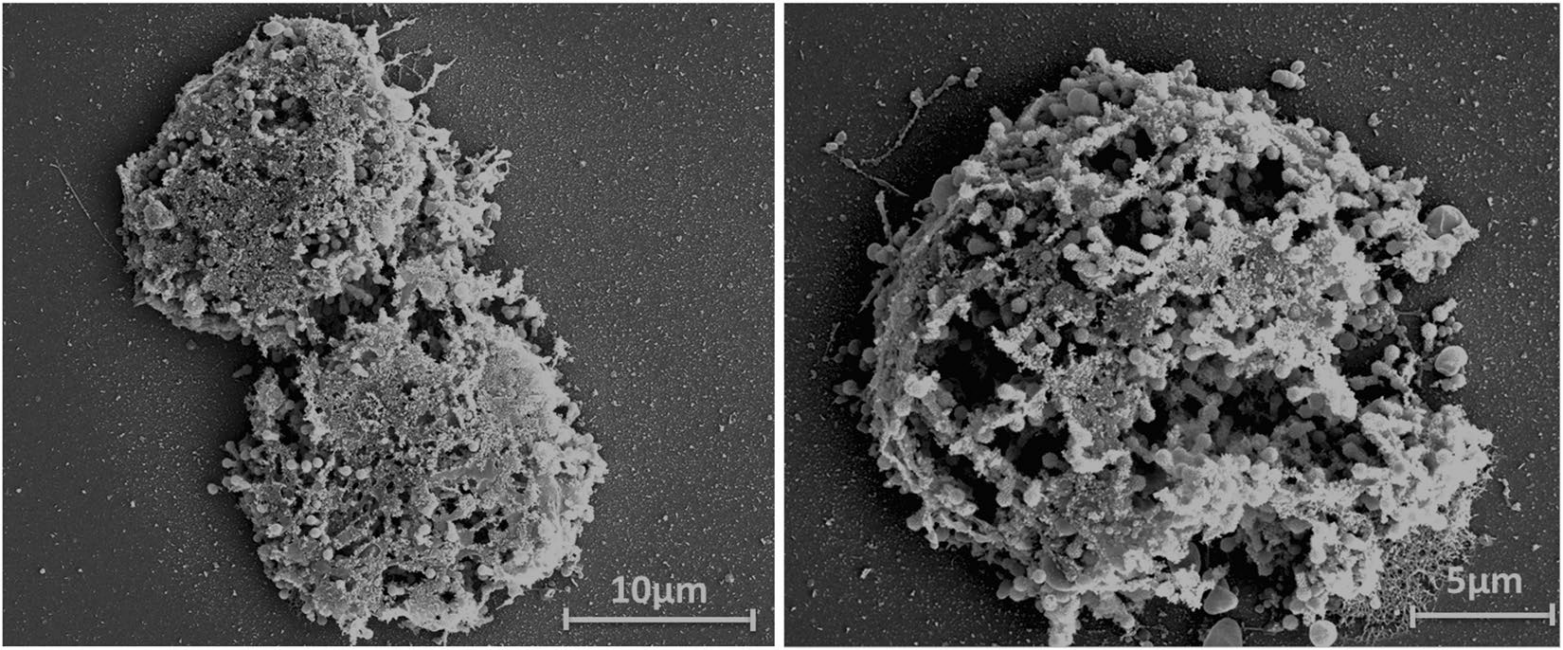
Late bunches are composed of highly damaged dead *A. castellanii* cells. *A. castellanii* cells shown intensely damaged at 24 h.p.i., as observed by SEM. Scale bars, 5 µm and 10 µm.

Another phenomenon that drew our attention was the occurrence of bunch reformation after mechanical breakdown (Fig. 4). Initially, we observed that early and late bunches could be easily disaggregated by homogenization using a pipette or by vortexing. However, 30 min after disaggregation, cells from early bunches (12 h.p.i.) were attracted to each other, forming large clusters of cells and assuming an appearance similar to early bunches before cell separation. In contrast, reformation of late bunches (24 h.p.i.) was not observed. In addition, using light microscopy we observed that some cells belonging to late bunches were larger in size, which was especially obvious after the disaggregation procedure (Fig. 4). The lack of bunch reformation at late timepoints indicates that at 24 h.p.i., cells were already killed by the viral infection, and a simple homogenization was able to permanently disaggregate the cells that formed late bunches. To confirm this hypothesis, we performed SEM on late bunches. Interestingly, we observed cells with the plasma membranes almost completely degraded and filled with intricate networks of tupanvirus particles and cell debris (Fig. 5).

Taken together, these results indicate that tupanvirus promotes the formation of large aggregates of amoebas, which are able to re-aggregate as long as cells are still alive. These findings motivated us to investigate possible factors that may interfere in bunch formation and the biological relevance of tupanvirus-promoted bunches.

### Amoebal-bunch formation correlates with increased mRNA levels of mannose binding protein genes

*Acanthamoeba* spp. is widely distributed in the environment and can cause human diseases, mainly associated with immunocompromised patients and contact lenses wearers. Furthermore, *Acanthamoeba* spp. has a relevant role in ecosystems, due to its ability to act as a host for microbial pathogens^20–23^. Mannose receptor proteins are well known for promoting interactions between amoebas and other cells. In this way, previous studies revealed that mannose-binding protein (MBP) present on the surface of *Acanthamoeba* is one of the major virulence proteins and may be essential for pathogenesis and invasion of host cells^11,15,24^ demonstrated that the potential of *Acanthamoeba* pathogenicity is directly dependent on the level of MBP^24^. In addition, previous studies showed that *A. castellanii* treated with mannose had reduced adhesion and cytotoxicity, strongly suggesting that its pathogenicity is associated with MBP^11,25^. Interestingly, after an in-depth analysis of the tupanvirus soda lake genome, we found that the virus has an 525 bp MBP gene. The domain involved in mannose recognition contains a three-fold internal repeat, and the consensus sequence motif is QXDXNXVXY. A previous study has suggested that the QDN subdomains may be essential for functionality of the protein. In addition, the importance of Y in subdomain of the MBP-mannose interaction was demonstrated^26^.

**Figure 6:**
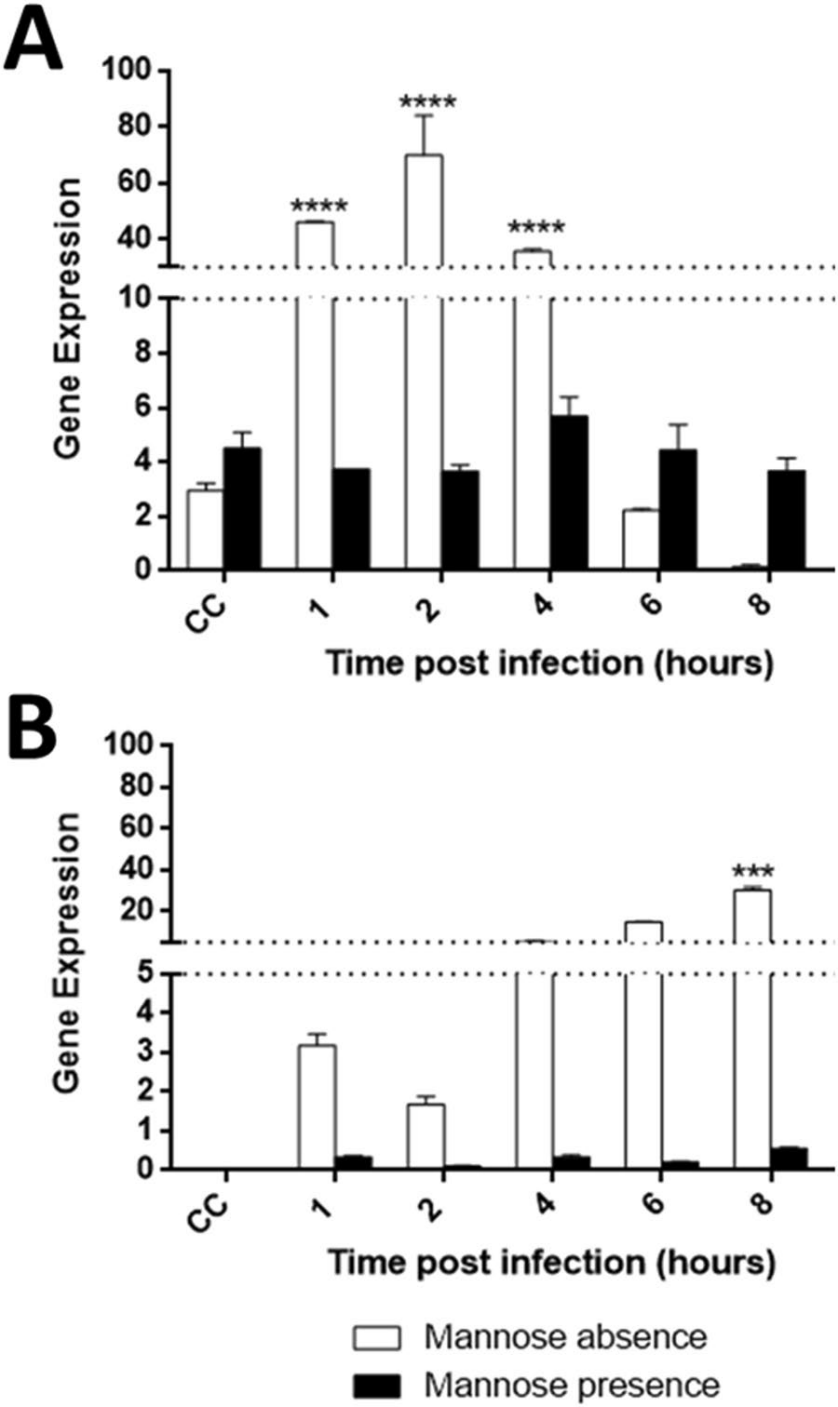
Tupanvirus upregulates the expression of viral and cellular mannose binding protein (MBP) genes. *A. castellanii* cells were infected with tupanvirus and collected at 1, 2, 4, 6 and 8 h.p.i. and MBP mRNA levels were measured by qPCR: cellular (**A**) and viral (**B**) genes with and without the presence of mannose. The data were calculated using the 2^−(Δct)^ method and are shown as the standard deviation of two independent biological assays. Error bars indicate SDs. The statistical significance was calculated using a two-tailed 2-way ANOVA test, Tukey’s range test, using GraphPad Prism. ****p < 0.0001.

This finding led us to investigate the MBP viral and cellular expression during the replication of tupanvirus and the possible effects of free mannose on the formation of bunches. An analysis of cellular MBP gene expression in uninfected *A. castellanii* cells showed a basal level of MBP transcripts, meaning it is likely essential for cellular processes (Fig. 6A). However, during tupanvirus infection, the levels of cellular MBP transcripts significantly increased (*p* = 0.0001) at earlier times during infection (1, 2 and 4 h.p.i.). The increase of cellular MBP gene expression induced by tupanvirus precedes bunch formation by a few hours (6 h.p.i.) (Figs 1A and 6A). Regarding tupanvirus encoding MBP, we observed a gradual increase in (or accumulation of) transcripts through the infection time-course. High levels of viral MBP transcripts were observed from 4 until 8 h.p.i (Fig. 6B). Therefore, our data indicate that the expression of cellular and viral MBP genes is induced during tupanvirus infection, suggesting a possible relevance of these genes in the viral replication cycle (Fig. 6).

Interestingly, orthologous MBP genes were found in the genome of other members of the *Mimiviridae* family, such as in the prototype APMV. Phylogenetic analysis of the amino acid sequence of viral MBP showed that tupanviruses forms an out-grouping among amoebal-mimiviruses (Suppl. Fig. 3A). Considering APMV does not induces the formation of bunches in infected amoebas, an analysis was performed to determine whether APMV was able to induce the expression of cellular MBP, as well as whether this virus express its own MBP gene throughout the time-course of infection. We observed that APMV induced a decrease in cellular MBP gene expression, even when compared to uninfected cells, in contrast to tupanvirus soda lake, which significantly increases cellular MBP gene expression which may be related to the induction of bunch formation (Suppl. Fig. 4B).

The expression of APMV MBP gene transcripts was also analyzed during the time-course of APMV infection in *A. castellanii*, revealing that this gene was significantly expressed at 6 and 8 h.p.i. (Suppl. Fig. 4A). However, an analysis of the APMV MBP amino acid sequence revealed polymorphisms in all three of the repeats that compose the catalytic site of the protein (the mannose-binding domain), more specifically in the QDN subdomains (Suppl. Fig. 3B), which have been associated with a loss of MBP function^26^. Polymorphisms in the Y amino acids have also been observed in the first repeat of the APMV MBP catalytic site (Suppl. Fig. 3B), which has also been associated with a loss of MBP function^26^. In contrast, tupanvirus MBP has no polymorphisms in any of the repeats in the catalytic site, when compared to the functional protein previously studied (Suppl. Fig. 3B)^26^. In summary, mutations in the mannose-binding domains of the APMV MBP gene could indicate that this protein is not able to bind to mannose, although more studies into structural level are needed to confirm this hypothesis.

**Figure 7:**
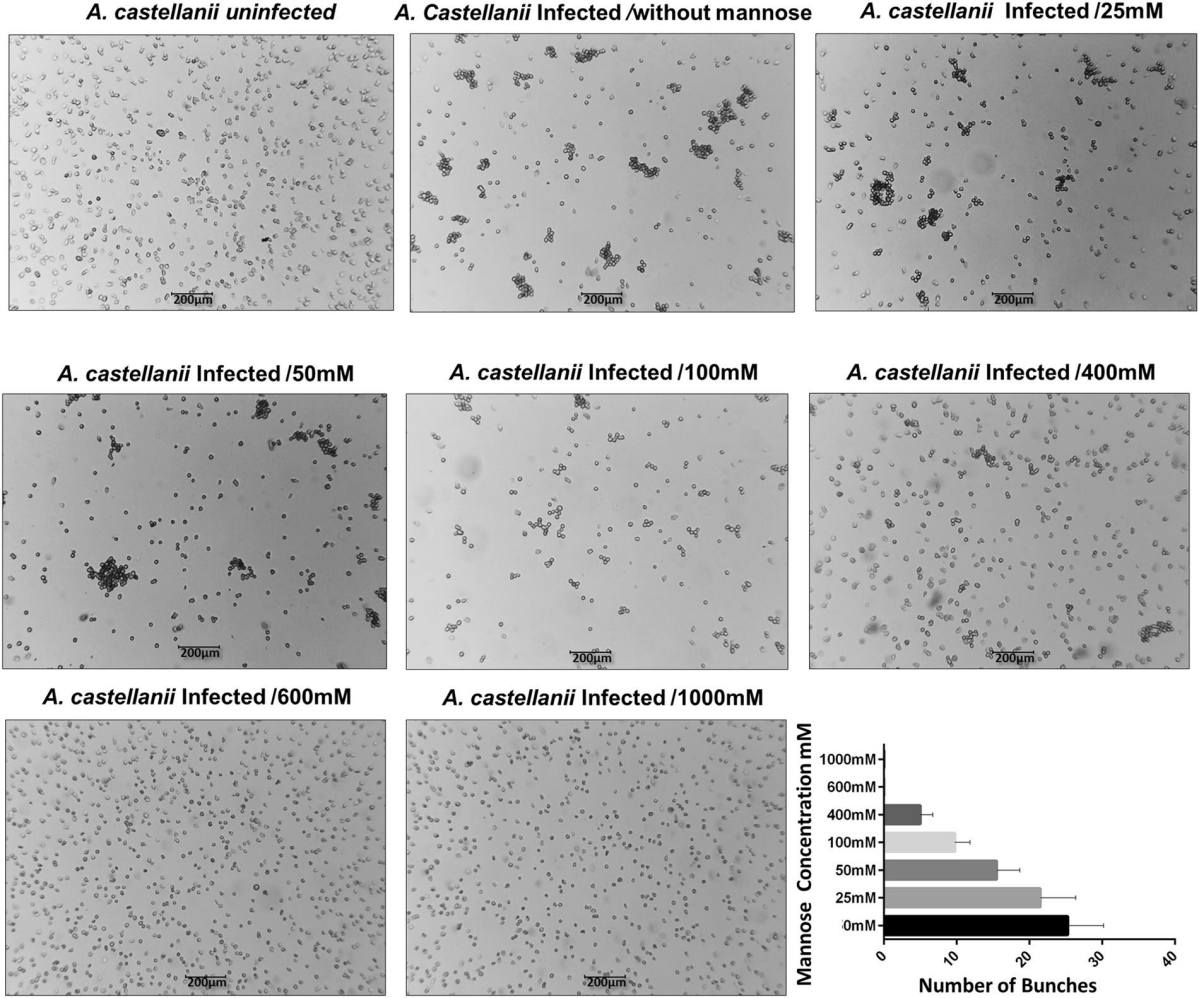
Free-mannose affects tupanvirus-induced bunch formation in a dose-dependent manner. *A. castellanii* cells were treated with free-mannose at concentrations ranging from 25 to 1000 mM in PAS solution, and then infected with tupanvirus (M.O.I. of 10). Eight hours post-infection, cells were observed by optical microscopy. As controls, *A. castellanii* cells cultivated in PAS medium without mannose and uninfected cells were cultured with 600 mM mannose. Bar graphs represent the number of bunches found per ml in different concentrations of mannose. Error bars indicate SDs. The treatment of amoebas with 600 mM mannose did not affect the replication of tupanviruses in amoebas and did not induced cytotoxic effects in uninfected *A. castellanii*. Scale bar, 200 µm.

Previous work have demonstrated that free-mannose negatively affects *Acanthamoeba* adhesion to cell surfaces and interferes with amoeba pathogenicity^16,25^. Therefore, the impact of free-mannose on cellular and viral MBP gene expression was here evaluated in uninfected and tupanvirus-infected cells. Our results showed that free mannose negatively affects the expression of the cellular MBP gene, maintaining it at close to basal levels (Fig. 6A). The same negative effect was observed in relation to the expression of the tupanvirus MBP gene (Fig. 6B). Considering these results, the effect of free mannose on the cytopathic effect of tupanvirus was evaluated as well. Interestingly, when *Acanthamoeba* was pre-treated with free-mannose, there was a gradual inhibition of bunch formation in a dose-dependent manner (Fig. 7), suggesting that formation of amoebal-bunches correlates with the suppression of viral and cellular MBP genes (Fig. 7).

Previous studies have shown that *Acanthamoeba* MBP is itself a glycoprotein containing mannose^15^, which may be one of the factors that explains the formation of the bunches in cells infected by tupanvirus, since this virus induces the expression of the MBP gene. Thus, the induction of mannose receptor genes in the tupanvirus replication cycle may be important for the optimization of the formation of bunches, since the interaction between amoebas may occur through interactions among their receptors.

### Bunch-forming cells are able to interact with uninfected cells

**Figure 8:**
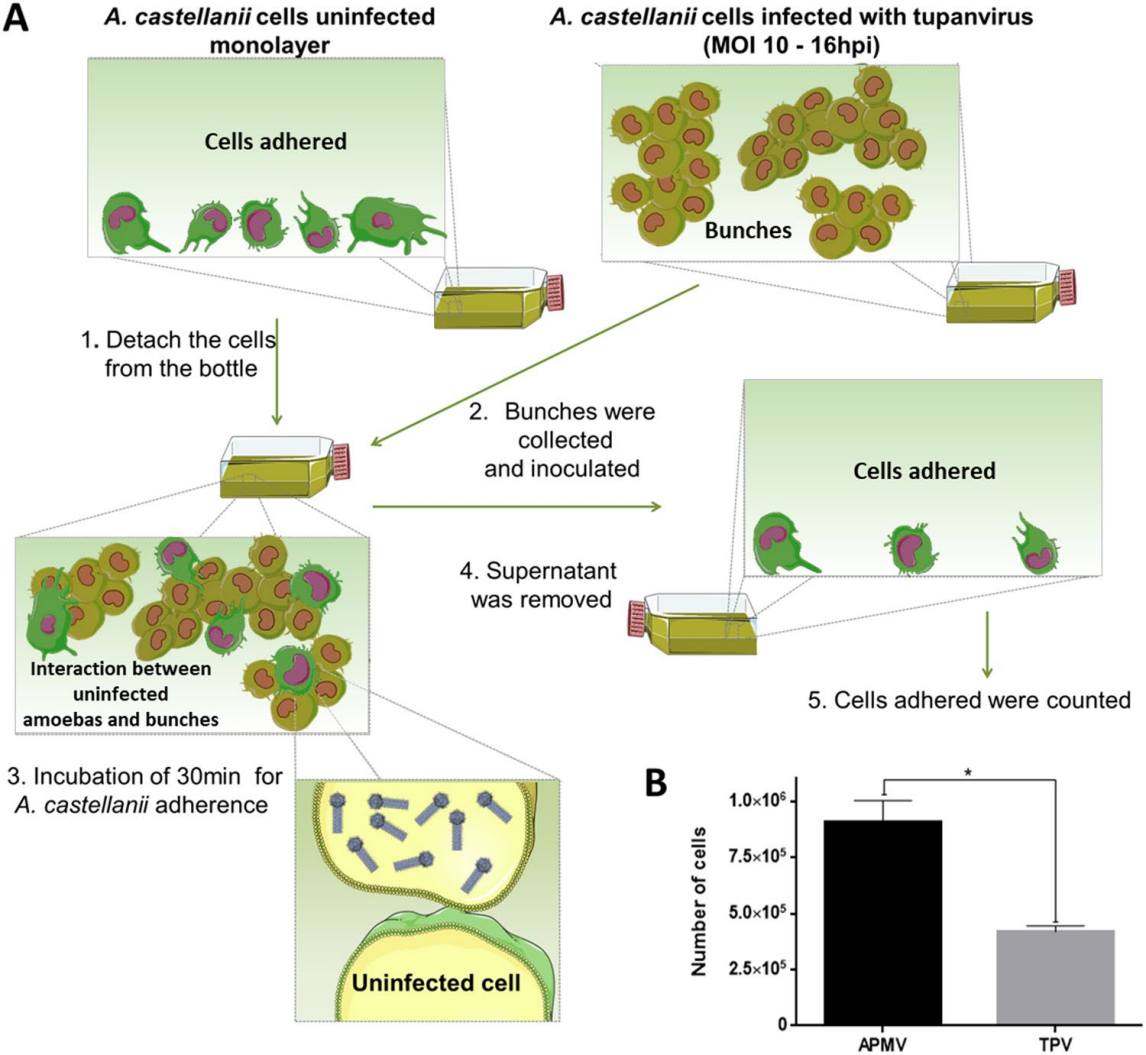
Tupanvirus-induced bunches may bind to and hijack uninfected cells. (**A**) *A. castellanii* cells were infected with tupanvirus at an M.O.I. of 10 and at 16 h.p.i. bunches were collected and used to inoculate flasks containing fresh, uninfected *A. castellanii* cells. After 30 min of incubation, the supernatant in flasks containing bunches and uninfected cells was removed. The remaining cells attached to the flask were then quantified. As a control, APMV-infected amoebae (which does not induce bunches formation) were used in parallel with tupanvirus-infected ones. (**B**) Remaining cells attached to flasks after incubation with tupanvirus-induced bunches or cells infected with APMV were then counted. Tupanvirus-induced bunches attached to and hijacked uninfected cells, causing a significant decrease of remaining adherent cells in the flasks, compared to APMV-infected cells. The statistical significance was calculated using a two-tailed Student’s t-test. *p < 0.05, performed using GraphPad Prism. Error bars indicate SDs.

As previously characterized in this study, the formation of the bunches starts at approximately 6 h.p.i. and persists until at least 24 h.p.i. At 16 h.p.i., almost all the cells are infected and clustered forming large bunches (Fig. 1A). An experiment was designed in order to evaluate whether bunch-forming cells were able to interact with uninfected cells (Fig. 8A). For this, *A. castellanii* cells were infected with tupanviruses (at an M.O.I. of 10), and at 16 h.p.i. the bunches were collected and used to inoculate a flask containing uninfected *A. castellanii* cells in suspension. After incubation, the supernatant (containing bunches and eventual non-infected amoebas interacting with bunches) were removed. The cells that remained adhered to the flask (the ones that did not interact with bunches) were counted to evaluate if the bunches were able to hijack the uninfected cells (Fig. 8). In addition, the supernatant was submitted to SEM and IF, using tupanvirus-specific antibodies. APMV-infected amoebae, which do not induce bunch formation, were used as a control in order to compare whether APMV-infected cells were also able to interact with and hijack uninfected cells (Fig. 8B).

**Figure 9:**
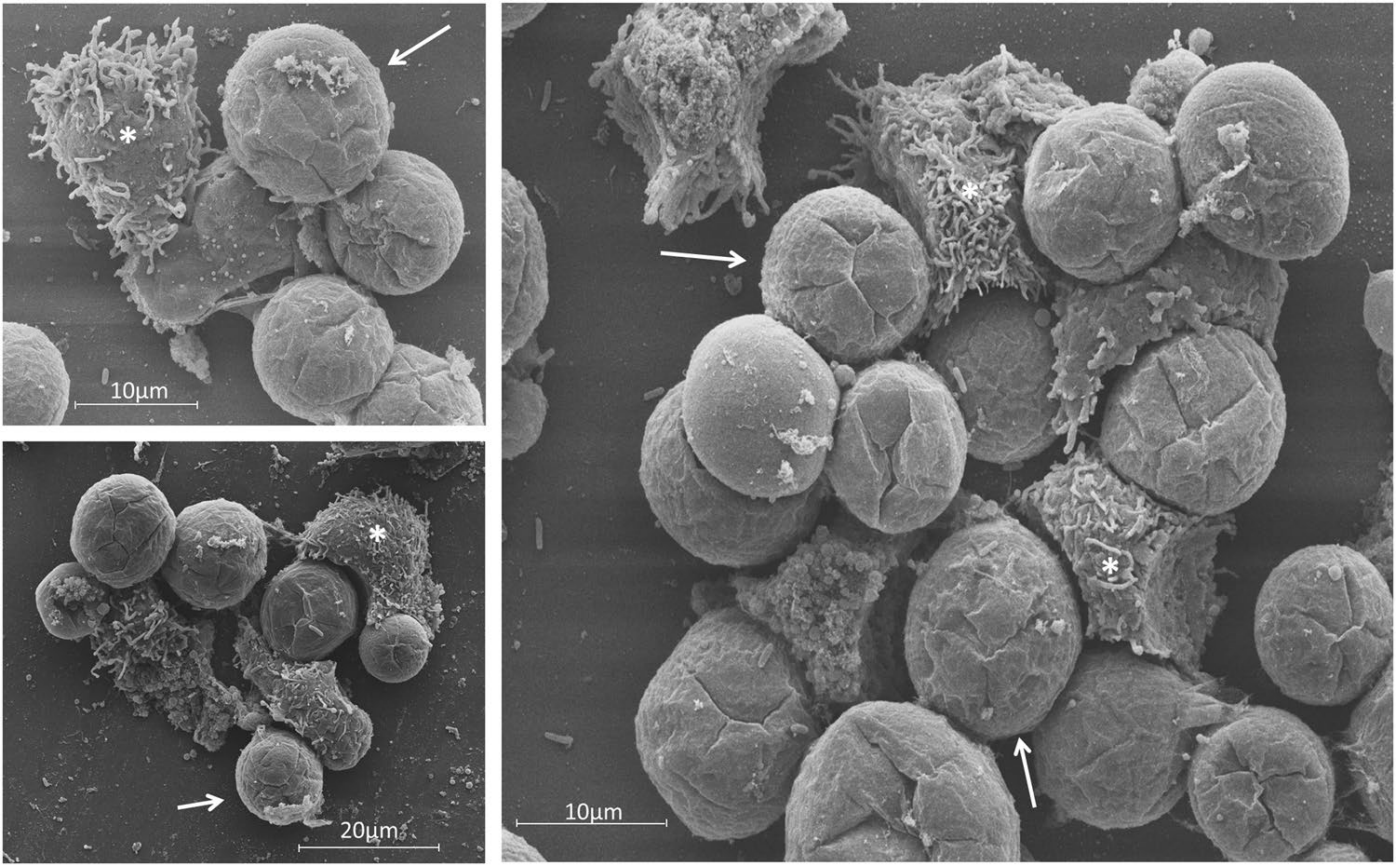
SEM showing the interaction between uninfected *A. castellanii* cells and tupanvirus-induced bunches. SEM images of uninfected cells that interacted with bunches and were carried by infected cells (experiment described in Fig. 8). Scale bars, 10 µm and 20 µm. Arrows indicate infected *A. castellanii* cells (rounded) and asterisks indicate uninfected *A. castellanii* cells (most with pseudopods).

Our results revealed that tupanvirus-induced bunches are able to bind to and hijack many uninfected cells. The number of cells remaining in the flasks after interaction with tupanvirus-induced bunches was significantly lower than those in flasks inoculated with APMV-infected cells (Fig. 8B). The interaction among tupanvirus-induced bunches and uninfected cells was also observed by SEM; it was possible to observe infected cells, which were rounded, interacting with fresh trophozoites presenting their typical pseudopods (Fig. 9). IF assays also showed bunches containing a mix of infected and uninfected cells (data not shown). Lastly, considering the ability of tupanvirus-induced bunches to bind and hijack uninfected cells, we evaluated whether such interactions could promote an increase in viral titer. For this, bunches were collected at 16 h.p.i. and inoculated in flasks containing fresh, uninfected amoebas, as previously described above. After incubation, the supernatant containing bunches attached to uninfected cells was collected and transferred to a new flask. Infectious particles in these population of cells were tittered at 0 and 36 h.p.i, revealing an of almost 2 log increase in viral titer (Suppl. Fig. 5).

Tupanviruses has the broadest host spectrum known among amoebal giant viruses^1^. While most of the giant viruses, such as cedratvirus, marseilleviruses, mollivirus, pandoraviruses, mimivirus, faustovirus and kaumoebavirus, are known to be able to replicate only in a single amoeba genus, tupanvirus is able to replicate in *Acanthamoeba* spp., *Vermoameba vermiformis*, *Dysctiostelium discoideum* and *Willartia magna*^1,5–9,27,28^. The generalist profile displayed by tupanviruses may be related to the extreme aquatic environments where they were isolated (high salinity, pH and depths); within inhospitable environments there is a lower species richness and abundance, meaning there are more hosts available and a better chance of a future infection^29^. In this way, bunch formation induced by tupanvirus may be important to the recruitment of cells, improving the chances of viral progeny to find a new host cell that may be scarce in those environments. In addition, bunch formation can reduce the dilution effect in aquatic environments, thereby allowing the release of more particles near the host, which could also facilitate the encounter between viral particles and their hosts.

## Concluding Remarks

Here we described a new virus-host interaction mechanism in which tupanvirus-infected amoebas are induced to aggregate with uninfected cells, forming bunches that can increases tupanvirus progeny. In addition, our results demonstrated that mannose reduced the transcript levels of cellular and viral mannose receptor genes, as well as inhibited bunch formation in a concentration-dependent manner, suggesting that amoebal-bunch formation correlates with MBP gene expression. Taken together, our results provide insights into the ecology of these intriguing viruses.

## Electronic supplementary material

Supplementary figures
