# Supplementary material for: Tupanvirus-infected amoebas are induced to aggregate with uninfected cells promoting viral dissemination
Source: Sci Rep. 2019 Jan 17;9:183. doi: 10.1038/s41598-018-36552-4 (PMC6336878; doi:10.1038/s41598-018-36552-4)
Supplement: Supplementary file 1 — Supplementary figures [file 41598_2018_36552_MOESM1_ESM.pdf]

Title: Tupanvirus-infected amoebas are induced to aggregate with uninfected cells promoting viral dissemination

Authors: Graziele Oliveira[1], Lorena Silva[1], Thiago Leão[1], Said Mougari[2], Flávio Guimarães da Fonseca[1]; Erna Geessien Kroon[1], Bernard La Scola[2], Jônatas Santos Abrahão[1*]

Authors affiliation: [1]*Departamento de Microbiologia, Instituto de Ciências Biológicas, Universidade Federal de Minas Gerais, Belo Horizonte, Minas Gerais, Brazil-* postal code 31270-901;[2] *URMITE, Aix Marseille Université, UM63, CNRS 7278, IRD 198, INSERM 1095, IHU - Méditerranée Infection, AP-HM, 19-21 boulevard Jean Moulin, 13005 Marseille, France*

***Corresponding author:** Jônatas Santos Abrahão, Departamento de Microbiologia, Universidade Federal de Minas Gerais, Belo Horizonte, Minas Gerais, Brazil, postal code 31270-901, +55 31 3409-3002, jonatas.abrahao@gmail.com.

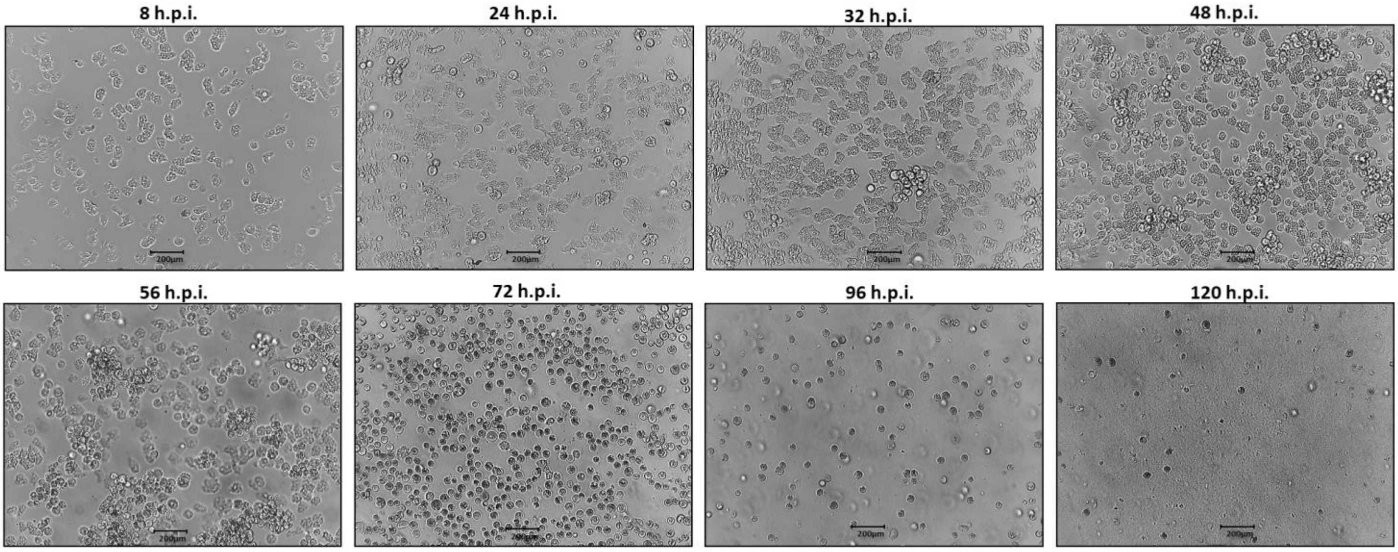

Suppl. Fig. 1. Characterization of tupanvirus' cytopathic effect at low M.O.I. *A. castellanii* infected at an M.O.I. of 0.01 showed rounding at approximately 24 h.p.i. At approximately 32 h.p.i., bunches were observed and become more evident at 48 and 56 h.p.i. At around 72 h.p.i., the disaggregation of bunches was observed. At late timepoints cell lysis (96 h.p.i.) and a large number of particles dispersed throughout the medium (120 h.p.i.) was observed. Scale bar, 200 µm.

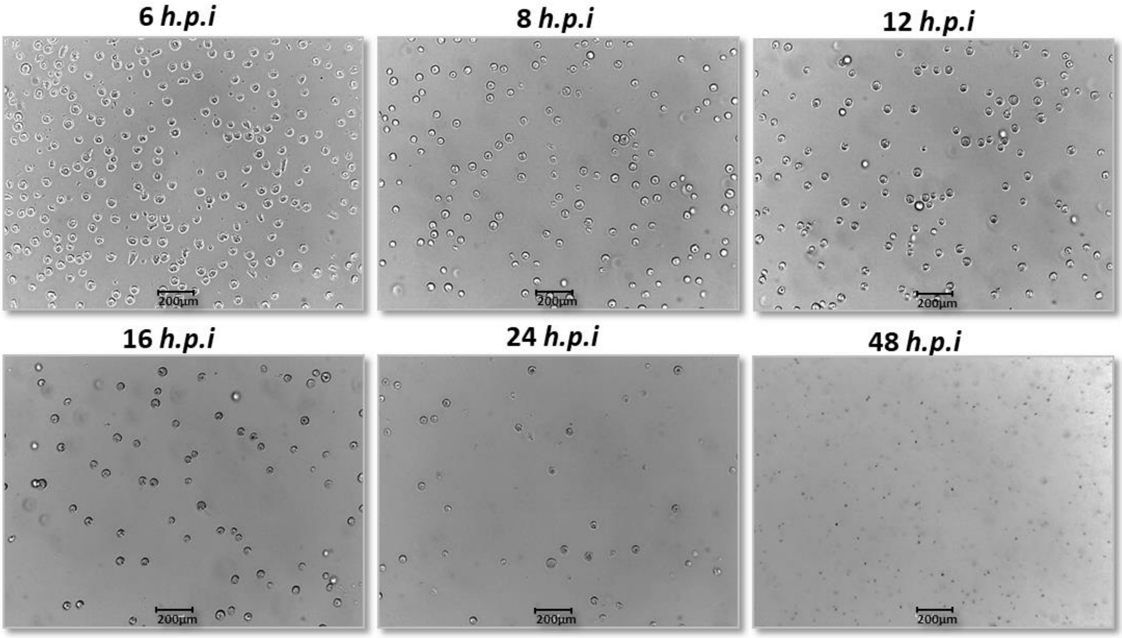

Suppl. Fig. 2. Characterization of APMV's cytopathic effect. APMV infected *A. castellanii* at an M.O.I. of 10 show rounding and cell lysis. Scale bar, 200 µm.

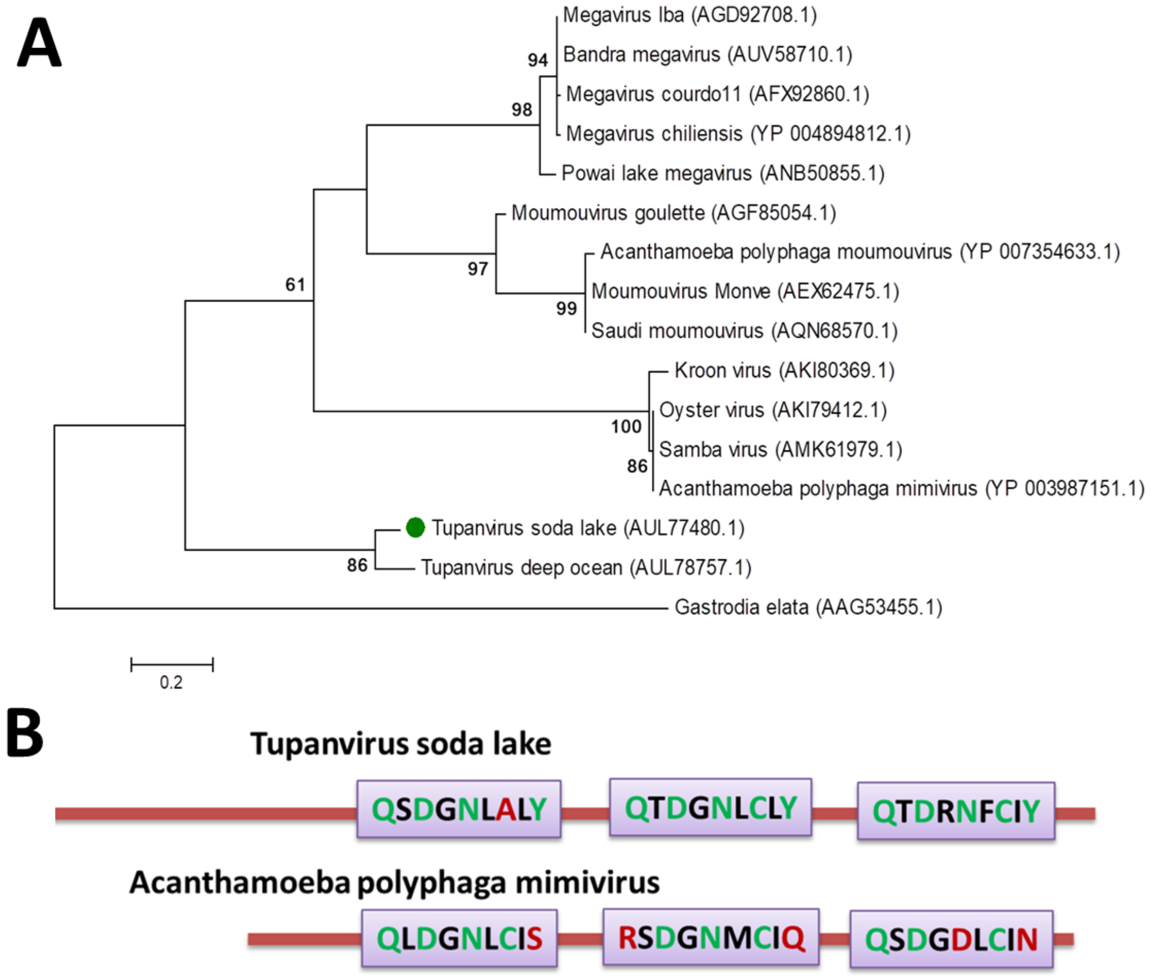

Suppl. Fig. 3. Phylogenetic reconstruction and domain comparison of the MBP. (A) MBP phylogenetic tree using the maximum likelihood method and based on amino acid sequences from tupanvirus and other representative members of the *Mimiviridae* family. The accession numbers are shown in the tree. (B) Schematic representation of tupanvirus and APMV MBP proteins, showing three repeats that compose the catalytic domain. The domain involved in mannose recognition contains a three-fold internal repeat, and the consensus sequence motif is QXDXNXVXY. The analysis of APMV MBP amino acid sequences revealed polymorphims in all three repeats that compose the catalytic site of the protein (mannose-binding domain), more specifically in the QDN subdomains, which were associated with a loss of MBP function. Polymorphism on the Y amino acids have also been observed in the first repeat of the APMV MBP catalytic site, which has also been associated with a loss of MBP function. In contrast, tupanvirus MBP protein has all repeats that would be expected to belong to a functional MBP catalytic site.

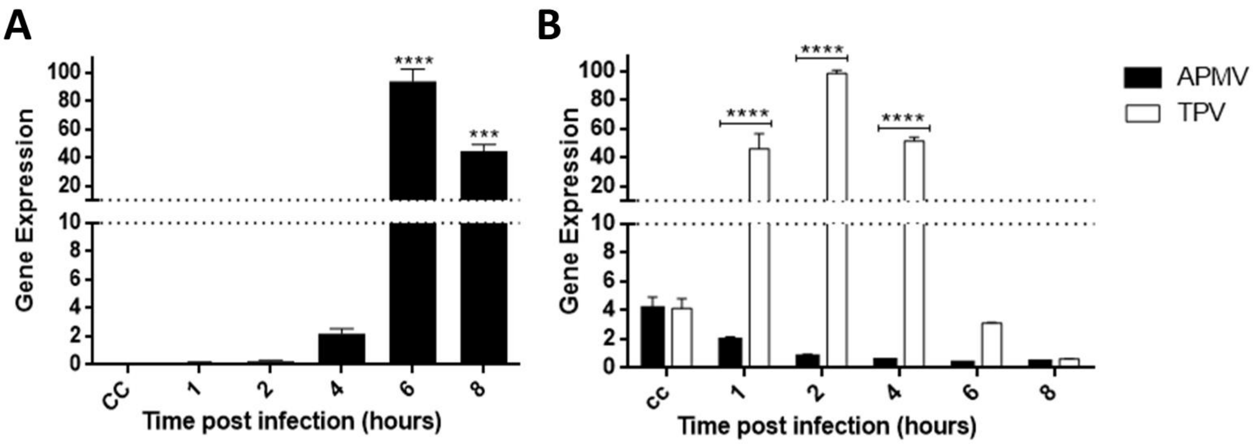

Suppl. Fig. 4. Analysis of the expression of viral (A) and cellular (B) mannose binding protein (MBP) mRNA level during APMV infection. *A. castellanii* cells were infected with APMV or tupanvirus at an M.O.I. of 10 and collected at 1, 2, 4, 6, 8 h.p.i. MBP mRNA levels were measured by qPCR. The data were calculated using the $2^{-(\Delta ct)}$ method and are represented as the standard deviation of two independent biological assays. Error bars indicate SDs. The statistical significance was calculated using one-way ANOVA-Dunnett's multiple comparisons test for analysis of the expression of viral MBP (A) and two-way ANOVA-Sidak's multiple comparisons test for analysis of the expression of cellular MBP (B) performed using GraphPad Prism software. *** $p < 0.001$; **** $p < 0.0001$.

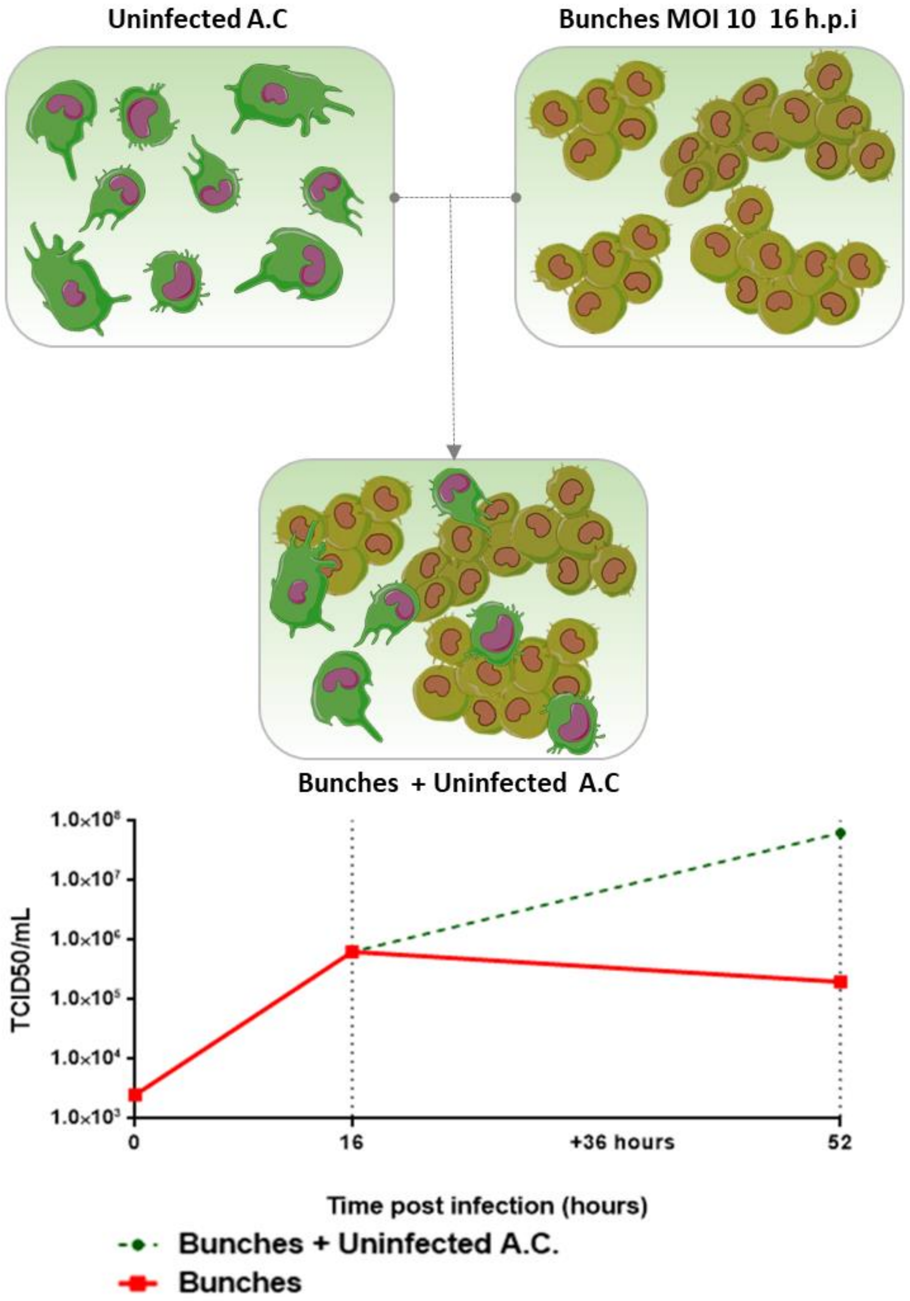

Suppl. Fig. 5. Interactions between bunches and uninfected cells promote an increase of tupanvirus progeny. *A. castellanii* cells were infected with tupanvirus at an M.O.I. of 10 and at 16 h.p.i. bunches were collected and used to inoculate flasks containing fresh uninfected amoebas, as previously described (Fig. 8). After incubation, supernatants in flasks containing bunches attached to uninfected cells were collected and transferred to a new flask. Infectious viral particles were tittered at 36 h.p.i., revealing an increase of almost 2 log in viral titers. Graph was constructed using GraphPad Prism.
